# Supplementary material for: A Pilot Study in Sweden on Efficacy of Benzylpenicillin, Oxytetracycline, and Florfenicol in Treatment of Acute Undifferentiated Respiratory Disease in Calves
Source: Antibiotics (Basel). 2020 Oct 26;9(11):736. doi: 10.3390/antibiotics9110736 (PMC7692533; doi:10.3390/antibiotics9110736)
Supplement: Supplementary file 1 [file antibiotics-09-00736-s001.pdf]

A Pilot Study in Sweden on Efficacy of Benzylpenicillin, Oxytetracycline, and Florfenicol in Treatment of Acute Undifferentiated Respiratory Disease in Calves

Virpi Welling [1], Nils Lundeheim [2] and Björn Bengtsson [3,*]

Supplementary Materials.

S1. Flow chart for the outcome of 117 calves enroled in the study.

Enroled calves: **117**
Farm S: 59
Farm H: 58

➡

2 calves did not fulfil the criteria for TEMP (a rectal temperature ≤39.5 °C and/or or a drop > 1 °C at 48 H after first treatment.
Farm S: 2
Farm H: 0

⬇

Remaining calves: **115**
Farm S: 57
Farm H: 58

➡

2 calves died within 30 days of first treatment
Farm S: 1
Farm H: 1

⬇

Remaining calves: **113**
Farm S: 56
Farm H: 57

➡

9 calves retreated at < 30 days
Farm S: 7*
Farm H: 2

⬇

Calves with a positive $RESP_{30}$ response: **104**
Farm S: 49
Farm H: 55

➡

4 calves retreated at 30-60 days
Farm S: 4**
Farm H: 0

⬇

Calves with a positive $RESP_{60}$ response: **100**
Farm S: 45
Farm H: 55

➡

5 calves retreated at >60 days - slaughter
Farm S: 5 ***
Farm H: 0

⬇

Calves with a positive $RESP_{tot}$ response: **95**
Farm S: 45
Farm H: 55

* Two calves were again retreated in the period 30-60 days; ** One calf was again retreated in the period <60 days - slaughter; *** one retreated calf died.

S2. Overview of the work on the study.

Design of study (October-December 2015)

- Non-inferiority study to compare efficacy of benzylpenicillin (PEN), oxytetracycline (OTC) or florfenicol (FLO), administered according to dosage regimens approved in Sweden, for treatment of BRD in calves.
- 3 herds to each treat 20 calves with PEN, 20 with OTC and 20 with FLO. In total 60 calves for each regimen.
- To detect a difference in efficacy of about 15% at a significance level of 5% with a statistical power of 80%.
- Calves semi randomly (consecutively) selected for treatment by farm personnel according to specified criteria and efficacy evaluated by farm personnel. Specified data registered by farm personnel and records kept at the farm.
- Ethical approval from Uppsala Försöksdjursetiska Nämnd was sought and received.

↓

Enrolment of herds (January 2016)

- Three farms specialised in raising calves purchased from dairy farms for slaughter were selected by convenience among the clients of one of the authors (VW). Criteria for selection were an appreciable number of calves contracting BRD and acceptance of the extra work involved in assessing calves and registering data.
- The three farms selected were contacted and later visited by the authors (VW, BB). At the visit, the background and design of the study were discussed in detail. Also, the carrying through of the study was presented in detail and farm personnel received written instructions.

↓

Carrying through of the study (February 2016–May 2017)

- Farm S carried out the study in the period February-April 2016 whereas the smaller farm H could not complete the collection of BRD cases until May 2017. For practical reasons, the third farm could not complete the study and dropped out in the spring of 2016.
- Data recorded on farm S and H were collected.

↓

Analysing and reporting the study (May 2017–September 2020)

- Collected data was checked and analysed. Additional data from slaughter (slaughter weight) was collected from slaughterhouses. (Since the last calf in the study was slaughtered in September 2019 the dataset was not complete until the autumn of 2019.)
- Writing and submission of manuscript.
